# Rare Mutations of *CACNB2* Found in Autism Spectrum Disease-Affected Families Alter Calcium Channel Function

**DOI:** 10.1371/journal.pone.0095579

**Published:** 2014-04-21

**Authors:** Alexandra F. S. Breitenkamp, Jan Matthes, Robert Daniel Nass, Judith Sinzig, Gerd Lehmkuhl, Peter Nürnberg, Stefan Herzig

**Affiliations:** 1 Department of Pharmacology, University of Cologne, Cologne, Germany; 2 Department of Child and Adolescent Psychiatry and Psychotherapy, LVR-Klinik Bonn, Bonn, Germany; 3 Department of Child and Adolescent Psychiatry and Psychotherapy, University of Cologne, Cologne, Germany; 4 Cologne Center for Genomics, University of Cologne, Cologne, Germany; 5 Center for Molecular Medicine, University of Cologne, Cologne, Germany; Emory University School Of Medicine, United States of America

## Abstract

Autism Spectrum Disorders (ASD) are complex neurodevelopmental diseases clinically defined by dysfunction of social interaction. Dysregulation of cellular calcium homeostasis might be involved in ASD pathogenesis, and genes coding for the L-type calcium channel subunits Ca_V_1.2 (*CACNA1C)* and Ca_V_β2 (*CACNB2*) were recently identified as risk loci for psychiatric diseases. Here, we present three rare missense mutations of *CACNB2* (G167S, S197F, and F240L) found in ASD-affected families, two of them described here for the first time (G167S and F240L). All these mutations affect highly conserved regions while being absent in a sample of ethnically matched controls. We suggest the mutations to be of physiological relevance since they modulate whole-cell Ba^2+^ currents through calcium channels when expressed in a recombinant system (HEK-293 cells). Two mutations displayed significantly decelerated time-dependent inactivation as well as increased sensitivity of voltage-dependent inactivation. In contrast, the third mutation (F240L) showed significantly accelerated time-dependent inactivation. By altering the kinetic parameters, the mutations are reminiscent of the *CACNA1C* mutation causing Timothy Syndrome, a Mendelian disease presenting with ASD. In conclusion, the results of our first-time biophysical characterization of these three rare *CACNB2* missense mutations identified in ASD patients support the hypothesis that calcium channel dysfunction may contribute to autism.

## Introduction

Autism spectrum disorder (ASD) is defined by dysfunction of social interaction and communication, stereotypic behavior and sensory integration problems. It is a complex neuro-developmental disease, which might result from an altered brain ontogenesis or altered neural homeostasis. According to the Centers for Disease Control and Prevention (CDC) the prevalence of ASD in the US population is 1∶88 [1]. Until now, genetic explanations for ASD are limited to rare chromosomal abnormalities like copy number variations or very rare single gene disorders. A first hint to unravel pathophysiological pathway of ASD came from the identification of the mutation p.G406R found in the L-type calcium channel pore-forming subunit (Ca_V_1.2) gene *CACNA1C* in patients with Timothy Syndrome (TS) [2]. Whole-cell patch clamp recordings showed that the TS-mutation leads to a decelerated and incomplete inactivation of the calcium inward current. The phenotype of TS demonstrates the consequences of inadequate inactivation behavior of voltage gated calcium channels (VGCC) in different biological contexts, like heart, brain, and the immune system. Calcium channel inactivation is a key mechanism by which cells are able to tightly control intracellular calcium levels and therefore the activity of excitable cells. Of note, calcium channel inactivation is not exclusively controlled by the channel pore, but also depends on auxiliary subunits, namely Ca_V_β and Ca_V_α2δ [3], [4]. The assembly of the subunits and their specific isoforms determines the distinctive behavior of VGCCs for the neuronal function. The number of permutations of calcium channel complexes with their subunits, isoforms and splice-variants paves the way for fine-tuned calcium channel function adapted for information processing. There are transcripts of ten pore-forming Ca_V_α1-subunit genes (CACNA1A to CACNA1I and CACNA1S), which undergo extensive alternative splicing and in concert with auxiliary subunits exhibit different biophysical properties and expression profiles as well as distinct subcellular targeting [5].

Particularly Ca_V_β-subunits have been shown to impact surface expression and modulation of channel activity and kinetics, leading to an increased L-type calcium channel activity, as revealed in whole-cell [6], [7] and single-channel recordings [8]. A wide variation of inactivation behavior has been described for the splice variants of the β2-subunit [4]. Even slight structural differences within the Ca_V_β-subunit can strongly modify the gating behavior of L-type calcium channels [8]–[10]. Interestingly, the expression of distinct Ca_V_β-subunits within the brain is dependent on the stage of neuronal development [11]. Since the discovery of the TS-mutation and its effect on inactivation of the L-type calcium channel, additional calcium channel genes or loci have been associated with ASD, e.g. the genes for the pore-forming subunits *CACNA1D* [12], *CACNA1F* [13], *CACNA1G* [14], *CACNA1H* [15] and the auxiliary subunit *CACNB1* [14]. These studies emphasize the importance of calcium channel activity, inactivation and calcium signaling in a broader sense for the pathophysiology of ASD. Interestingly, the activity and kinetics of several pore-forming subunits of L-type (Ca_V_1.1–1.4) and Non-L-type (Ca_V_2.1–2.3) channels [2], [12], [13], [16] are regulated by Ca_V_β-subunits. Moreover, they are involved in various signaling pathways [2], [17], [18], all of which have been linked to ASD pathophysiology previously. Nevertheless, studies considering the role of calcium channels in ASD have focused on the pore-forming subunits of the calcium channel complex, even though gene clusters of interacting proteins participating in linear signaling pathways would have a similar chance of being involved in the etiology of ASD [19].

Based on our previous studies showing that current and gating kinetics profoundly depend on the particular Ca_V_β-subunit isoform or splice variant associated with the channel pore [8], [9], we hypothesized that Ca_V_β-subunit mutations might lead to electrophysiological phenotypes similar to that observed in TS. Though all four Ca_V_β-subunit genes (*CACNB1*–*CACNB4*) are expressed within the brain [20] we chose *CACNB2* because of positional evidence from a meta-analysis of linkage data [21]. Here, Trikalinos and colleagues showed genome-wide suggestive significance for a designated bin 10p12–q11.1, which embraces a large genomic region including the *CACNB2* gene in autistic siblings. Recently, *CACNB2* was found as a risk locus for five major psychiatric disorders including ASD [22] and thus is regarded as a susceptibility gene.

In our current study, we searched for mutations in the β2-subunit gene that might affect gating behavior of voltage-dependent calcium channels. In a mutation screening, 155 patients with ASD were sequenced and the results were filtered for most promising candidates. We included only missense variants, which were not present in our cohort of 259 matched controls and located in highly conserved regions of the protein as an indicator of functional importance. Furthermore, the mutations had to be unknown or potentially damaging according to in-silico predictions.

Here, for the first time, we present three missense mutations located in conserved regions of the calcium channel *CACNB2* gene found in three families affected by ASD. In electrophysiological analyses of recombinant channels, these missense mutations were found to differentially alter current kinetics. Though our data do not prove an association between these *CACNB2* mutations and ASD, our findings support the idea of Cavβ2 variants being of functional relevance for ASD pathophysiology.

## Materials and Methods

### Ethics Statement

Procedures were approved by the Institutional Review Board (application number 04-223) of the medical faculty of the University of Cologne.

### Subjects

gDNA samples were available from 259 healthy controls and 155 patients with Autistic Spectrum Disorder (ASD), each are ethnically matched groups of Caucasians of west-European origin. The cases were elicited from three different sources: Twenty patients were recruited by the Department of Child & Adolescent Psychiatry and Psychotherapy, University of Cologne. These patients meet diagnostic criteria for ‘broad ASD’ (e.g. Aspergers’s) based on interview for DSM-IV-R. A written informed consent was obtained from the parents or legal guardians of the minors using the consent form approved by the I.R.B. of the University of Cologne (Germany). Ten cases were included from Autism Tissue Program (www. atpportal.org) from the Maryland National Institute of Child Health and Human Development (NICHD) Brain Tissue Center and the Harvard Brain Tissue Resource Center (USA), published e.g. in [23]. 125 cases were provided by the Autism Genetic Resource Exchange (AGRE) Consortium (http://research.agre.org/program/descr.cfm). All AGRE-Patients met diagnosis for ‘autism’ and each family was ascertained on the condition that at least two individuals were diagnosed with ASD [24], where diagnostic tests included autism diagnostic interview-revised (ADI-R) [25] and the autism diagnostic observational schedule (ADOS) [26]. Regulatory review, approval, and oversight of AGRE’s human subject research is provided by Western IRB (title: AUTISM GENETIC RESOURCE EXCHANGE). In order to find new sequence variations with a putative function-altering effect, all exons and their flanking intronic sequences of *CACNB2* were sequenced in 155 ASD patients. The number of sequenced cases enabled the detection of rare SNPs (f = 0.01) with a probability of 96% to detect it once and 81% to detect it twice. The power calculation was made according to Glatt et al. [27]. To assure a strong genetic background, patients from multiplex families with a severe phenotype were selected.

The control group consisting of 259 matching controls was kindly provided by Dr. Heusch and Dr. Brodde, published in [28]. Based on the gender-dependent frequency of ASD [1], we chose an appropriate male to female ratio for both groups of about 5∶1.

### Genotypic and DNA Sequence Analyses

Oligonucleotides to all known exons of the *CACNB2* gene were designed according to genomic sequences found in the Celera data base using Primer3. PCR amplification of DNA samples was carried out with GoTaq (Promega) according to the manufacturer’s protocol; annealing temperature was chosen according to Primer3. Mutational analyses were carried out with Mutation Surveyor. PCR fragments were purified using ExoI and SAP (NEB), and sequencing was performed with an ABI 3730 automated DNA sequencer.

### DNA Constructs and Site-directed Mutagenesis

For functional analysis, mutations p.G167S and p.S197F were introduced in human β_2d_cDNA (NM_201596.2) and the mutation p.F240L was introduced in β_2d_E7c_ (NM_201597.2) by site-directed mutagenesis (Stratagene QuikChange Kit) and verified by sequencing.

EGFP was used as reporter gene, which was coexpressed together with the β2-subunit by the bicistronic pIRES2-EGFP vector (Clontech). G167S forward primer 5′-gatagggcgattggtaaaagaaagctgtgaaatcggattc-3′; G167S reverse primer 5′-gaatccgatttcacagctttcttttaccaatcgccctatc-3′; S197F forward primer 5′-cagagagccaagcaagggaaattctacttcagtaaatcaggaggaaattcatcatcc-3′; S197F reverse primer 5′-ggatgatgaatttcctcctgatttactgaagtagaatttcccttgcttggctctctg-3′; F240L forward primer 5′-ggaaaactgcaggcttgctttggcggtttactaca; F240L reverse primer 5′-tgtagtaaaccgccaaagcaagcctgcagttttcc.

### Cell Culture

In brief, HEK-293α1c cells stably expressing Ca_v_1.2 subunit (GeneBank accession #NM_000719) cloned from human heart [29] were grown in Petri dishes in Dulbeccos modified Eagle medium (PAA, Germany) supplemented with 10% FBS (PAA, Germany), penicillin (10 U/ml), and streptomycin (10 µg/ml; PAA, Germany). Cells were selected by geneticin (G418) (PAA, Germany) at a final concentration of 500 µg/ml. Cells were routinely passaged twice a week and incubated at 37°C under 6% CO_2_ growth conditions. HEK-293 α1c cells were transfected using SuperFect reagent (Qiagen). For whole-cell recordings, cells were transfected with a 2∶3 ratio of either human WT or mutant β_2d_-subunit and human α_2_δ_1_-subunit [30].

### Electrophysiology

Whole-cell recordings in EGFP-positive cells were obtained 48–72 h after transfection. Immediately prior to recording, cells kept in 35-mm culture dishes were washed at room temperature (19–23°C) with bath solution. For constructs based on human β_2d_cDNA (NM_201596.2) the bath solution contained (in mM) 20 BaCl_2_, 1 MgCl_2_, 10 HEPES, 40 TEA-Cl, 10 dextrose, and 65 CsCl (pH 7.2 with TEA-OH) and pipette solution (in mM) 105 CsCl, 25 TEA-Cl, 11 EGTA, and 10 HEPES (pH 7.2 with CsOH). Holding potential was −100 mV. For constructs based on human β2d_E7c cDNA (NM_201597.2) the bath solution contained (in mM) 5 BaCl_2_, 1 MgCl_2_, 10 HEPES, 40 TEA-Cl, 10 dextrose, and 92 CsCl (pH 7.2 with TEA-OH). Patch pipettes made from borosilicate glass (1.7 mm diameter and 0.283 mm wall thickness, Hilgenberg GmbH, Malsfeld, Germany) were pulled using a Sutter Instrument P-97 horizontal puller and fire-polished using a Narishige MF-83 microforge (Narishige Scientific Instrument Lab, Tokyo, Japan). Pipette resistance was 2–4 MΩ.

Currents were elicited by applying test potentials of −40 mV to +60 mV from a holding potential of −100 mV using Clampex software pClamp 5.5 and an Axopatch 1D amplifier (Axon Instruments, Foster City, CA, USA). Voltage dependence of Ba^2+^ current inactivation in HEK cells was determined with a two-pulse protocol. For β_2d_cDNA (NM_201596.2) constructs the conditioning first pulse (stepped from −100 mV to +40mV) was held for 5s and the second pulse (of +10mV) for 125 ms; for β_2d_E7c_cDNA (NM_201597.2) constructs the conditioning pulse was stepped from −75 mV to +5 mV for 1 s and the second pulse (of +10 mV) was held for 125 ms. For both steady-state inactivation protocols the relative magnitude of inward current elicited during the second pulse was plotted as a function of the voltage of the conditioning first pulse.

Data were analyzed using pCLAMP6 (Axon Instruments) and GraphPad 5 Prism. Currents were filtered at 2 kHz. Data were fitted to a Boltzmann function to obtain the half point (V0.5) and slope factor (dV) for the voltage dependence of inactivation; Fits were performed after subtracting the offset from the peak values of the steady-state inactivation data. Offset was defined as a deviation from zero at the end of a fully inactivating current (after a 5000ms prepulse followed by a 125ms test pulse at +10mV). For voltage dependence of activation data were fitted by combined Ohm and Boltzmann relation 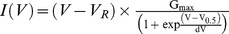 according to [31].

### Statistical Analysis

Student’s unpaired two-tailed t-test (GraphPad Prism 5, GraphPad Software, San Diego, CA) was used to compare electrophysiological parameters gained with the several CACNB2 mutations and their corresponding wildtypes. P<0.05 was considered significant. All p-values are listed in table S2. Data are presented as mean ± SEM.

## Results

**Figure 1:**
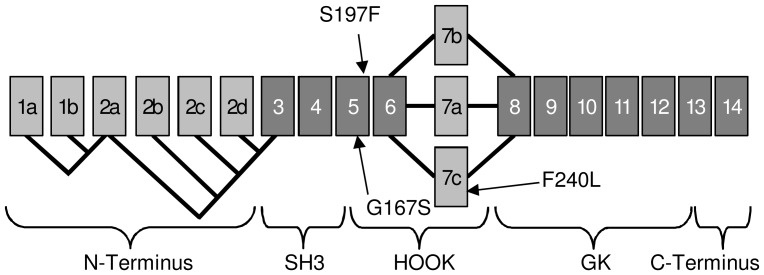
Splice scheme of human *CACNB2* resulting in 9 splice variants of the Ca_V_β2-subunit with the localization of the three mutations. Spliced exons are shown in light grey and conserved exons in dark grey. All nine splice variants express the mutation-carrying exon 5, while three of variants contain the localization of the third mutation in exon 7c [10].

**Figure 2:**
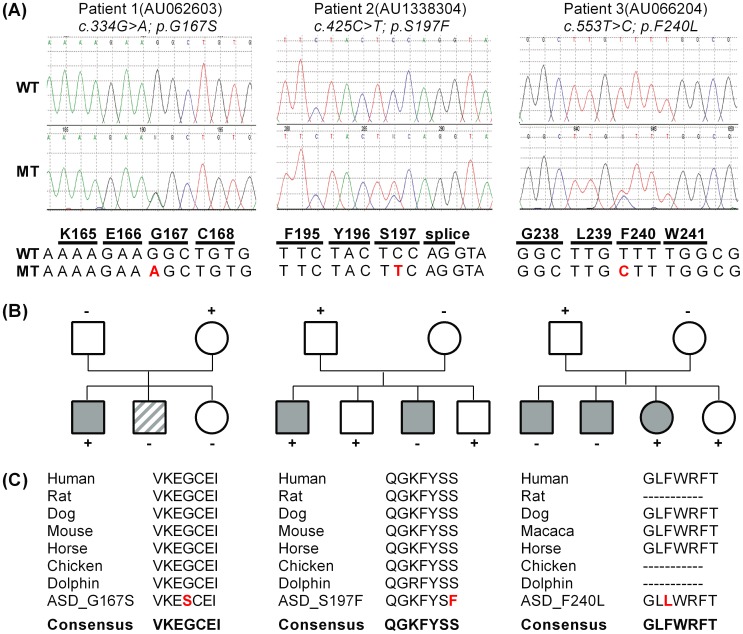
(**a**) Chromatogram showing point mutations for patient 1–3. (**b**) The pedigrees show the occurrence of the disease phenotype and the inheritance of missense mutations in *CACNB2*. *Circles* and *squares* indicate females and males, respectively. *Filled* symbols denote diagnosis of autism; *partially filled* symbols denote individuals that are diagnosed within a broad spectrum of pervasive developmental disorders. (**c**) Alignments show conservation among different species for p.G167S, p.S197F and p.F240L suggesting an importance for the topology and function.

We performed a screening for mutations in *CACNB2* by sequencing all 19 exons and their flanking intronic regions from 155 patients with ASD. All variants found are listed in table S1. We filtered for variants causing non-synonymous substitutions, frameshifts, or splice site changes and identified six missense mutations. Since two of them occurred at similar frequencies in our patient cohort, our control cohort, and the NCBI database of short genetic variations (dbSNP), they were not further considered. One substitution (“New11” in tabl S1.) is likely to be benign according to the prediction of polymorphism phenotyping tool (PolyPhen2); also the amino acid exchange of the minor allele is prevalent in other species (data not shown). Three non-synonymous mutations were located in conserved domains of the protein (Fig. 1) and subsequently analyzed for functional effects. They were detected in three different patients who all were heterozygous carriers (Fig. 2a). The mutations were absent from 259 healthy controls.

Patient 1 showed a variation at cDNA position 334 from guanine to adenine resulting in an amino acid exchange from glycine to serine (p.G167S, referring to NM_201596.2). The substitution is not annotated in dbSNP and according to the PolyPhen2 prediction ‘probably damaging’ for the Ca_V_β2 function. Patient 2 had the cytosine at position 425 changed to thymine resulting in a substitution of phenylalanine for serine (p.S197F). This mutation is an annotated rare variant and shows a ‘possibly damaging’ PolyPhen2 score. The third affected patient had the transition c.T553C which results in a phenylalanine-to-leucine change (p.F240L, referring to NM_201597.2). The missense mutation is not annotated and PolyPhen2 predicts a ‘benign’ phenotyping score. The mutations p.G167S and p.S197F were found by analyzing the fifth exon, which is expressed in all splice isoforms; p.F240L was found in the alternative exon 7c (nomenclature according to Foell et al. [32]). All patients were diagnosed with autism by both ADI-R and ADOS. The clinical features of the patients are compiled in table 1.

**Table 1:** Clinical Features of ASD patients with rare mutations in *CACNB2*.

Description	Patient 1	Patient 2	Patient 3
	(AU062603)	(AU1338304)	(AU066204)
**Mutations**	*c.334G>A*	*c.425C>T*	*c.553T>C*
*(# NM_201597.2)*	*p.G167S*	*p.S197F*	*p.F240L*
**Genotype**	*+–*	*+–*	*+–*
**Gender**	M	M	F
**Epilepsy**	Yes	Unknown	No
Type	Complex partial	–	–
**Autism**			
ADI-R	Autism	Autism	Autism
ADOS	Autism	Autism	Autism
**Brain Imaging**			
MRI	normal	Unknown	Normal

**ADI-R**, Autism Diagnostic Interview-Revised; **ADOS**, Diagnostic Observation Schedule-Generic; **MRI,** magnetic resonance imaging.

The subsequently performed cosegregation analysis showed a low penetrance. Two variants showed incomplete segregation since some siblings, who were not obviously affected, were carriers of the mutation (Fig. 2b). Since complex-genetic disorders manifest depending on multiple factors, a low penetrance and incomplete cosegregation can be expected. In the literature, it has been suggested that pathogenic variants might be masked by epigenetic marks and thus are less penetrant [33] or that pathogenic variants follow an oligogenic pattern of inheritance, resulting in more severe or different phenotypes or are modified by the presence of additional variants [34]. Accordingly, unaffected carriers of a mutation might be possibly influenced by unknown genetic and epigenetic mechanisms [35] or might be subclinically affected.

As members of the membrane-associated guanylate kinase (MAGUK) protein family [8]–[10], β-subunits are comprised of two highly conserved central (GK and SH3) domains, flanked and interspersed by more variable N- and C-termini and a central HOOK domain (Fig. 1). The β2-subunit is subject to extensive alternative splicing of the N-terminus and within the central HOOK domain, comprising the alternative exons 7a-c (Fig. 1) [36]. All three mutations are located within the HOOK domain (Fig. 1), which is known to modulate calcium channel inactivation [37], [38]. The wildtype sequence of all three missense mutations revealed full conservation across species (Fig. 2c) indicating functional relevance.

To investigate the effect of the mutations on calcium channel function, we heterologously expressed WT and mutant forms of the human β2 together with human α2δ1 and EGFP in HEK-293 cells stably expressing a human Ca_V_1.2 pore-forming subunit, followed by whole-cell patch-clamp recordings. There are nine splice-variants of Ca_V_β2, with different inactivation kinetics [4]. The mutations p.G167S and p.S197F are present in all these Ca_V_β2 splice forms, and there is no predominant splice form in the brain. In accordance with our hypothesis and deductions from the TS-channel model, we expect a reduction of the channels inactivation rate. To evaluate this effect in the mutations we performed whole-cell experiments using Ba^2+^ as charge carrier, since the TS-mutation selectively decelerates time-dependent inactivation and spares or even accelerates calcium-dependent inactivation [39]. For the quantification of mutants’ influence on channel inactivation behavior we chose the β2d splice variant as a suitable reference isoform, because of its relatively fast inactivation kinetics [4], [8]. We compared the WT with the mutations p.G167S and p.S197F and the mutation p.F240L was analyzed separately with an appropriate WTE7c including its alternative exon 7c. The respective splice forms β2d (NM_201596.2) and β2dE7c (NM_201597.2) are – according to the GNF Expression Atlas 1 and 2 - expressed in fetal brain and in various adult brain regions.

**Figure 3:**
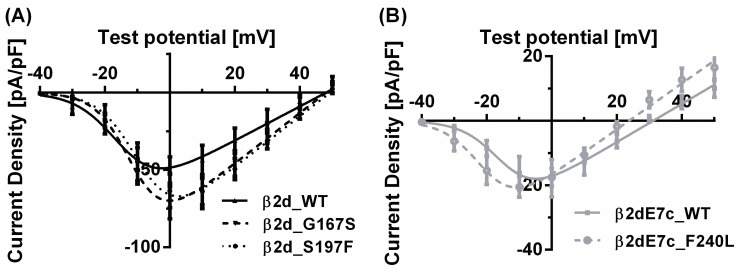
I–V relationships. Currents were elicited from −40 to +60 mV in 10 mV increments with 5 mM or 20 mM Ba^2+^, respectively. The holding potential was −100 mV. Current density of the variants p.G167S (N = 11) and p.S197F (N = 8) in (**a**) and p.F240L (N = 5) in (**b**) were compared with their respective WTs (β2d_WT: N = 9 and β2dE7c_WT: N = 5).

Peak current densities tended to be enhanced in the mutants β2d_G167S and β2d_S197F compared to β2d_WT. These mutations also caused a slight, non-significant shift of the I-V relationships towards more positive test potentials (Fig. 3a). Conversely, for the mutation of the β2dE7c_WT (β2dE7c_F240L), the I-V relationship revealed a trend towards enhanced channel activity at negative test potentials (Fig. 3b).

**Figure 4:**
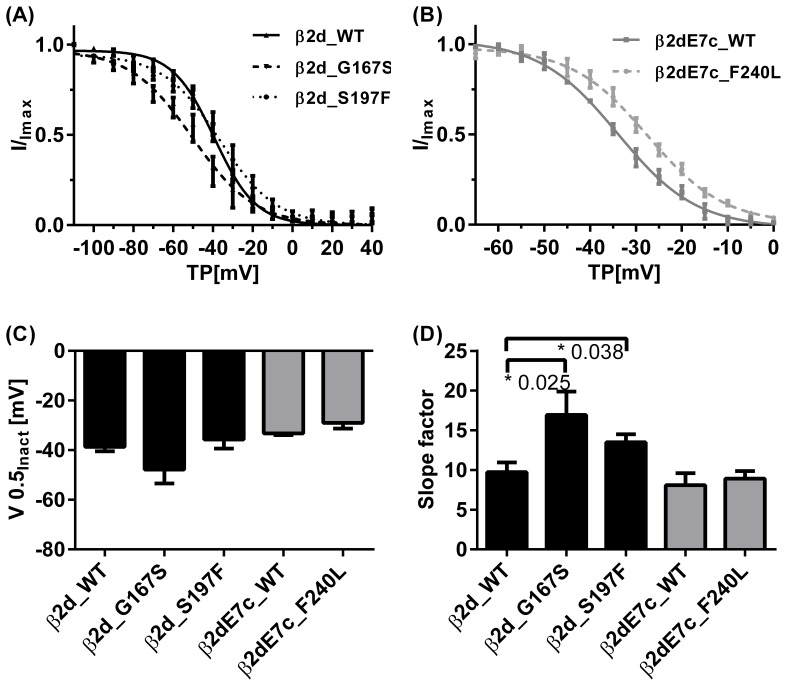
Voltage-dependent steady-state inactivation (a, b) of Ba^2+^ currents through L-type calcium channels. The ASD mutants p.G167S (N = 4) and p.S197F (N = 6) showed a significantly flattened slope of voltage-dependent inactivation compared to β2d_WT (N = 7). The third mutation β2dE7c_F240L. (N = 5) did not obviously differ from its corresponding β2dE7c_WT (N = 2). Half-inactivation potentials (V0.5_inact_) (**c**) and the slope factors dV (**d**) were obtained from the fits of individual experiments using the Boltzmann equation and averaging the results. Asterisk (*) marks a statistical significance (P<0.05) compared to the respective WT.

Steady-state inactivation was examined using two-pulse protocols. Data and Boltzmann fits are depicted in Fig. 4a and 4b. The voltage of half-maximal inactivation (V0.5_inact_) did not differ significantly between the three mutant channel complexes and their respective controls (Fig. 4c). However, a significant flattening of the Boltzmann curves was observed for β2d_G167S and β2d_S197F, as indicated by the slope factor dV (Fig. 4d). Numeric values are compiled in table 2.

**Table 2:** Current and voltage parameters (mean ± SEM) of the constructs tested.

	−β2d	β2d_WT	β2d_G167S	β2d_S197F	β2dE7c_WT	β2dE7c_F240L
	N = 3	N = 9	N = 11	N = 8	N = 5	N = 5
Peak Current Density [pA/pF]	−1.2	−56.5	−72.3	−68.8	−17.8	−21.1
	±1.0	±8.6	±10.3	±9.7	±5.8	±3.6
V0.5_act_ [mV]	–	−11.9	−9.8	−6.3	−13.6	−18.7
		±2.0	±1.2	±2.5	±0.36	±2.5
**Steady-state Inactivation**		**N = 7**	**N = 4**	**N = 6**	**N = 2**	**N = 5**
V0.5_inact_ [mV]		−38.7	−47.9	−35.7	−33.3	−28.2
		±1.8	±5.6	±3.7	±0.7	±1.9
dV		9.7	16.9 *	13.5 *	8.1	8.8
		±1.2	±2.9	±1.0	±1.5	±0.7

**V0.5_act_,** Voltage of half-maximal activation; **V0.5_inact_,** half-maximal inactivation voltage, dV, slope factor; −**β2d,** mock transfected cells;

*indicates P<0.05 versus corresponding WT.

**Figure 5:**
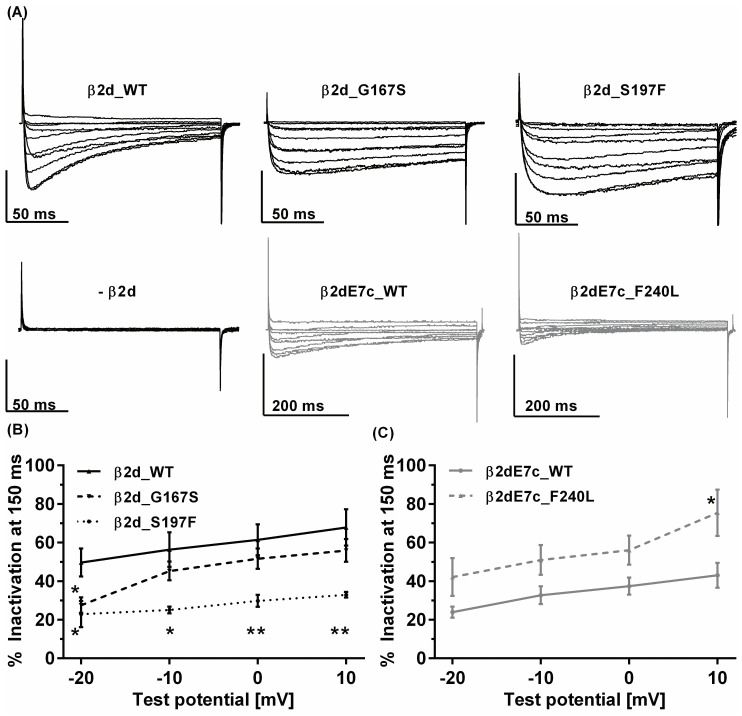
Time-dependent inactivation. Representative whole-cell Ba^2+^ current traces (a) of Ca_V_1.2/α2δ1 co-expressed with β2d_WT -subunit or co-expressed with the ASD mutants β2d_G167S, β2d_S197F or the β2dE7c_WT or its variant β2dE7c_F240L. The currents were elicited from a holding potential of −100 mV by 150 ms (or 500ms) step depolarization of −40 to +50 mV with 20 mM Ba^2+^ (for β2d variants) or 5 mM Ba^2+^ (for β2dE7c variants), respectively. Analysis of the extent of time-dependent inactivation. (**b**, **c**) % Inactivation was analyzed as the remaining fraction of whole-cell current that has not inactivated after 150 ms of depolarization. β2d_WT (N = 12), β2d_G167S (N = 8), β2d_S197F (N = 11) (**c**) Similar analysis for β2dE7c variants β2dE7c_WT (N = 5), β2dE7c_F240L (N = 6). Asterisk (*) marks a statistical significance (P<0.05) compared to the respective WT, (**) marks P<0.01.

As described previously for the Timothy Syndrome, the most notable differences between mutants and WT were observed in the time course of whole-cell Ba^2+^ currents. The mutant β2d_G167S and β2d_S1967F subunit induced a decelerated time-dependent inactivation of the channel complex within a 150ms test pulse (Fig. 5a). The WT with the alternative exon 7c inactivated more slowly than the WT with exon 7a. It was therefore examined using an extended test pulse duration (see scale bars in Fig. 5a). Compared with the adequate β2dE7c_WT the mutant β2dE7c_F240L showed an accelerated time-dependent inactivation. The mutants and the WT with Exon 7a were recorded with 20mM Ba^2+^ (compare [9]) and those with exon 7c were recorded with 5mM Ba^2+^ because of insufficient voltage-control with 20 mM Ba^2+^ and prolonged test pulse protocols (500ms). We analyzed the extent of time-dependent inactivation as the percentage of current that has inactivated after 150ms of depolarization (% inactivation). As expected from the representative whole-cell current traces, the currents inactivated significantly more slowly for all test potentials in case of β2d_S197F and at −20mV in case of β2d_G167S (Fig. 5b). In contrast, the mutant β2d_F240L led to faster, more pronounced inactivation compared to its corresponding WT (Fig. 5c). After 1000ms the currents of β2d_WT, β2d_G167S and β2d_S197F showed a comparable extent of inactivation of 94±1%, 99±2% and 96±3% at 0mV test potential.

## Discussion

The complex genetic principle of origin underlying ASD is still to be elucidated [40]. So far, genome-wide association and linkage studies presented inconsistent loci, reflecting a broad etiological heterogeneity and suggesting the influence of rare variants weighted by common susceptibility alleles. To put the spectrum and the genes into a pathophysiological context, an oligogenic model with epistasis has been assumed [41], [42]. It describes - based on their level of biological function - a combination of multiple interacting genes resulting in ASD phenotypes.

Until now, few potential pathophysiological mechanisms have been postulated [43], [44]. The role of impaired calcium channel inactivation for ASD was revealed by Splawski and colleagues with the TS-mutation (*CACNA1C*: p.G406R) [2]. In more general terms, calcium signaling was found by a gene pathway analysis based on the ‘Kyoto Encyclopedia of Genes and Genomes’ (KEGG) as one of the three most significant pathways for ASD [45]. The concept is further supported by the association of mutations in various calcium channel genes with non-idiopathic ASD [2], [13]–[16]. The activity and regulation of all L-type and some non-L-type calcium channels are dependent on their auxiliary β-subunit; therefore, the highly differentiated β-subunit function is expected to be crucial for proper function of the calcium channel complex and homeostasis in the nervous system.

Here, we present for the first time rare substitutions in highly conserved residues of the calcium channel subunit β2 in three out of 155 individuals with ASD, all of which were absent from 259 matching controls. According to the exome variant server (http://evs.gs.washington.edu/EVS), 100 variations (35 of which are missense, splice or frame-shift mutations) are known for European Americans in *CACNCB2*. Thus, a statistical proof of an elevated frequency of rare variants in the *CACNB2* gene in ASD patients was considered not feasible. We did not test for differences in the mutation frequency between ASD patients and controls. Instead, we searched for most promising candidates of putative causal variants. Subsequently, by using patch clamp, the variants were tested for putative impairment of the function of the channel complex. Electrophysiological analyses in HEK-cells by whole-cell patch-clamp recordings demonstrate that all three missense mutations significantly alter the kinetics of the currents carried by the Ca_V_1.2 complex. Two mutations in the Ca_V_β2-subunit lead to deceleration of time-dependent inactivation of Ba^2+^ currents as well as altered sensitivity of voltage-dependent inactivation. The third mutation shows a non-significant hyperpolarizing shift in current-voltage relation and an accelerated time-dependent inactivation. Of note decelerated time-dependent inactivation and incomplete voltage-dependent inactivation behavior are the biophysical hallmarks of the TS-mutation p.G406R in *CACNA1C* [2], [17], [39].

We observed a low penetrance of the three mutations in the ASD families under investigation, indicating the influence of other factors on the full expression of the condition. However, rare variants might contribute to the complex genetics and clinical heterogeneity of ASD. For comparison, an analogous sequencing approach studying *CACNA1H* as a candidate gene for ASD in 461 patients revealed six non-synonymous mutations in conserved domains, all showing a low penetrance and incomplete segregation [15]. Expression of complex genetic disorders depends on multiple factors, therefore a low penetrance of causal variants is quite common. Unaffected carriers might be subclinically affected, other risk factors might have contributed to the phenotype in the affected individuals, or the described mutations may only act as modifiers of the phenotype. Even for the Timothy syndrome - one of the most penetrant monogenic forms of autism - ASD has a penetrance of ∼75% only [46].

The putative effects of the Ca_V_β2-mutations are ample as the β-subunit also plays an essential role as an interaction partner with intracellular signaling machineries. For instance, Krey et al. have recently demonstrated a new pathway involving the TS-mutation which leads to a calcium channel activity-dependent and conformation-dependent dendrite retraction via Gem-induced RhoA-signaling [18]. Here, the Ca_V_β-subunits might be involved in two ways: firstly they are known to regulate the channel activity, but secondly the interaction of Ca_V_1.2 with Gem, mediated through the β-subunit is important for the ability of the channel to control activity-dependent dendritic arborization [18]. In summary, the role of the Ca_V_β2-subunit as a risk factor for ASD can not only be attributed to its interaction with diverse ASD-associated pore-forming Ca_V_α1-subunits, but also because the Ca_V_β2-subunit acts as a signaling hub and can link together different ASD pathways.

The mutations presented here appear to follow a similar but milder mechanism of action that occurs in TS. TS presents with a multi-organ dysfunction, possibly indicating the consequences of a dramatic elevation of the intracellular calcium concentration. Mutations resulting in an even larger effect would likely abort the organism’s development [47]. Thus the mutation of the TS can be viewed as an extreme of the viable spectrum of mutations within the calcium signaling pathway. Compared to this, the mutations β2d_G167S, β2d_S197F and β2d_F240L exhibited rather moderate effects on channel gating that nonetheless might suffice to unbalance neuronal calcium channel function.

Because private and rare mutations seem to play a role in the predisposition to ASD [44], the discovery of rare variants with putative functional relevance might contribute to our understanding of the disorder’s etiology. Integrating the data from TS [2], the meta-analysis of psychiatric disorders [22], and functional studies on auxiliary β-subunits, we propose that inappropriate function of different components of the voltage-gated calcium channel complex can result in or may contribute to autism spectrum disorder. More detailed biophysical and cell-biological studies under physiological conditions are warranted for all such mutations.

## Supporting Information

Table S1 **All found variants in the exons and flanking intronic regions of CACNB2 in autistic patients.**
(DOC)

Table S2 **Current and voltage parameters (mean ± SEM) of the constructs tested. P-values stem from individual Student t-tests of the indicated channels.**
(DOC)

## References

[1] Centers for Disease Control and Prevention (2012) Prevalence of autism spectrum disorders–Autism and Developmental Disabilities Monitoring Network, 14 sites, United States, 2008. MMWR Surveill Summ 61: 1–19. 22456193

[2] Splawski I, Timothy KW, Sharpe LM, Decher N, Kumar P, et al (2004) Ca(V)1.2 calcium channel dysfunction causes a multisystem disorder including arrhythmia and autism. Cell 119: 19–31. 15454078 10.1016/j.cell.2004.09.011

[3] Birnbaumer L, Qin N, Olcese R, Tareilus E, Platano D, et al (1998) Structures and functions of calcium channel beta subunits. J Bioenerg Biomembr 30: 357–375. 9758332 10.1023/a:1021989622656

[4] Takahashi SX, Mittman S, Colecraft HM (2003) Distinctive modulatory effects of five human auxiliary beta2 subunit splice variants on L-type calcium channel gating. Biophys J 84: 3007–3021. 12719232 10.1016/S0006-3495(03)70027-7 PMC1302863

[5] Ludwig A, Flockerzi V, Hofmann F (1997) Regional expression and cellular localization of the alpha1 and beta subunit of high voltage-activated calcium channels in rat brain. J Neurosci 17: 1339–1349. 9006977 10.1523/JNEUROSCI.17-04-01339.1997 PMC6793722

[6] Jangsangthong W, Kuzmenkina E, Khan IF, Matthes J, Hullin R, et al (2009) Inactivation of L-type calcium channels is determined by the length of the N terminus of mutant beta(1) subunits. Pflugers Arch 459: 399–411. 19821165 10.1007/s00424-009-0738-z

[7] Cens T, Mangoni ME, Nargeot J, Charnet P (1996) Modulation of the alpha 1A Ca2+ channel by beta subunits at physiological Ca2+ concentration. FEBS Lett 391: 232–237. 8764980 10.1016/0014-5793(96)00704-1

[8] Herzig S, Khan IF, Grundemann D, Matthes J, Ludwig A, et al (2007) Mechanism of Ca(v)1.2 channel modulation by the amino terminus of cardiac beta2-subunits. FASEB J 21: 1527–1538. 17289923 10.1096/fj.06-7377com

[9] Jangsangthong W, Kuzmenkina E, Khan IF, Matthes J, Hullin R, et al (2010) Inactivation of L-type calcium channels is determined by the length of the N terminus of mutant beta(1) subunits. Pflugers Arch 459: 399–411. 19821165 10.1007/s00424-009-0738-z

[10] Buraei Z, Yang J (2010) The b subunit of voltage-gated Ca2+ channels. Physiol Rev 90: 1461–1506. 20959621 10.1152/physrev.00057.2009 PMC4353500

[11] McEnery MW, Vance CL, Begg CM, Lee WL, Choi Y, et al (1998) Differential expression and association of calcium channel subunits in development and disease. J Bioenerg Biomembr 30: 409–418. 9758336 10.1023/a:1021997924473

[12] O’Roak BJ, Vives L, Girirajan S, Karakoc E, Krumm N, et al (2012) Sporadic autism exomes reveal a highly interconnected protein network of de novo mutations. Nature 485: 246–250. 22495309 10.1038/nature10989 PMC3350576

[13] Hemara-Wahanui A, Berjukow S, Hope CI, Dearden PK, Wu SB, et al (2005) A CACNA1F mutation identified in an X-linked retinal disorder shifts the voltage dependence of Cav1.4 channel activation. Proc Natl Acad Sci U S A 102: 7553–7558. 15897456 10.1073/pnas.0501907102 PMC1140436

[14] Strom SP, Stone JL, Ten BoschJR, Merriman B, Cantor RM, et al (2010) High-density SNP association study of the 17q21 chromosomal region linked to autism identifies CACNA1G as a novel candidate gene. Mol Psychiatry 15: 996–1005. 19455149 10.1038/mp.2009.41 PMC2889141

[15] Splawski I, Yoo DS, Stotz SC, Cherry A, Clapham DE, et al (2006) CACNA1H mutations in autism spectrum disorders. J Biol Chem 281: 22085–22091. 16754686 10.1074/jbc.M603316200

[16] Limpitikul W, Johny MB, Yue DT (2013) Autism-Associated Point Mutation in CaV1.3 Calcium Channels alters their Regulation by Ca2+ [Abstract]. Biophys J 104.

[17] Splawski I, Timothy KW, Decher N, Kumar P, Sachse FB, et al (2005) Severe arrhythmia disorder caused by cardiac L-type calcium channel mutations. Proc Natl Acad Sci U S A 102: 8089–8096 discussion 8086–8088. 15863612 10.1073/pnas.0502506102 PMC1149428

[18] Krey JF, Pasca SP, Shcheglovitov A, Yazawa M, Schwemberger R, et al (2013) Timothy syndrome is associated with activity-dependent dendritic retraction in rodent and human neurons. Nature Neuroscience 16: 201–209. 23313911 10.1038/nn.3307 PMC3568452

[19] Iossifov I, Zheng T, Baron M, Gilliam TC, Rzhetsky A (2008) Genetic-linkage mapping of complex hereditary disorders to a whole-genome molecular-interaction network. Genome Res 18: 1150–1162. 18417725 10.1101/gr.075622.107 PMC2493404

[20] Volsen SG, Day NC, McCormack AL, Smith W, Craig PJ, et al (1997) The expression of voltage-dependent calcium channel beta subunits in human cerebellum. Neuroscience 80: 161–174. 9252229 10.1016/s0306-4522(97)00115-2

[21] Trikalinos TA, Karvouni A, Zintzaras E, Ylisaukko-oja T, Peltonen L, et al (2006) A heterogeneity-based genome search meta-analysis for autism-spectrum disorders. Mol Psychiatry 11: 29–36. 16189507 10.1038/sj.mp.4001750

[22] Cross-Disorder Group of the Psychiatric Genomics Consortium (2013) Identification of risk loci with shared effects on five major psychiatric disorders: a genome-wide analysis. The Lancet 381: 1371–1379. 10.1016/S0140-6736(12)62129-1 PMC3714010 23453885

[23] Lintas C, Altieri L, Lombardi F, Sacco R, Persico AM (2010) Association of autism with polyomavirus infection in postmortem brains. J Neurovirol 16: 141–149. 20345322 10.3109/13550281003685839

[24] Geschwind DH, Sowinski J, Lord C, Iversen P, Shestack J, et al (2001) The autism genetic resource exchange: a resource for the study of autism and related neuropsychiatric conditions. Am J Hum Genet 69: 463–466. 11452364 10.1086/321292 PMC1235320

[25] Lord C, Rutter M, Le Couteur A (1994) Autism Diagnostic Interview-Revised: a revised version of a diagnostic interview for caregivers of individuals with possible pervasive developmental disorders. J Autism Dev Disord 24: 659–685. 7814313 10.1007/BF02172145

[26] Lord C, Risi S, Lambrecht L, Cook EH Jr, Leventhal BL, et al (2000) The autism diagnostic observation schedule-generic: a standard measure of social and communication deficits associated with the spectrum of autism. J Autism Dev Disord 30: 205–223. 11055457

[27] Glatt CE, DeYoung JA, Delgado S (2001) Service SK, Giacomini KM, et al (2001) Screening a large reference sample to identify very low frequency sequence variants: comparisons between two genes. Nat Genet 27: 435–438. 11279528 10.1038/86948

[28] Leineweber K, Bogedain P, Wolf C, Wagner S, Weber M, et al (2007) In patients chronically treated with metoprolol, the demand of inotropic catecholamine support after coronary artery bypass grafting is determined by the Arg389Gly-beta 1-adrenoceptor polymorphism. Naunyn Schmiedebergs Arch Pharmacol 375: 303–309. 17541557 10.1007/s00210-007-0166-6

[29] Schultz D, Mikala G, Yatani A, Engle DB, Iles DE, et al (1993) Cloning, chromosomal localization, and functional expression of the alpha 1 subunit of the L-type voltage-dependent calcium channel from normal human heart. Proc Natl Acad Sci U S A 90: 6228–6232. 8392192 10.1073/pnas.90.13.6228 PMC46901

[30] Schleithoff L, Mehrke G, Reutlinger B, Lehmann-Horn F (1999) Genomic structure and functional expression of a human alpha(2)/delta calcium channel subunit gene (CACNA2). Genomics 61: 201–209. 10534405 10.1006/geno.1999.5941

[31] Karmazinova M, Lacinova L (2010) Measurement of cellular excitability by whole cell patch clamp technique. Physiol Res 59 Suppl 1S1–7. 10.33549/physiolres.932000 20626213

[32] Foell JD, Balijepalli RC, Delisle BP, Yunker AM, Robia SL, et al (2004) Molecular heterogeneity of calcium channel beta-subunits in canine and human heart: evidence for differential subcellular localization. Physiol Genomics 17: 183–200. 14762176 10.1152/physiolgenomics.00207.2003

[33] Feinberg AP (2010) Genome-scale approaches to the epigenetics of common human disease. Virchows Arch 456: 13–21. 19844740 10.1007/s00428-009-0847-2 PMC3107986

[34] Mitchell KJ (2011) The genetics of neurodevelopmental disease. Curr Opin Neurobiol 21: 197–203. 20832285 10.1016/j.conb.2010.08.009

[35] Geschwind DH (2011) Genetics of autism spectrum disorders. Trends Cogn Sci 15: 409–416. 21855394 10.1016/j.tics.2011.07.003 PMC3691066

[36] Colecraft HM, Alseikhan B, Takahashi SX, Chaudhuri D, Mittman S, et al (2002) Novel functional properties of Ca(2+) channel beta subunits revealed by their expression in adult rat heart cells. J Physiol 541: 435–452. 12042350 10.1113/jphysiol.2002.018515 PMC2290333

[37] Miranda-Laferte E, Schmidt S, Jara AC, Neely A, Hidalgo P (2012) A short polybasic segment between the two conserved domains of the beta2a-subunit modulates the rate of inactivation of R-type calcium channel. J Biol Chem 287: 32588–32597. 22851179 10.1074/jbc.M112.362509 PMC3463332

[38] He LL, Zhang Y, Chen YH, Yamada Y, Yang J (2007) Functional modularity of the beta-subunit of voltage-gated Ca2+ channels. Biophys J 93: 834–845. 17496037 10.1529/biophysj.106.101691 PMC1913152

[39] Barrett CF, Tsien RW (2008) The Timothy syndrome mutation differentially affects voltage- and calcium-dependent inactivation of CaV1.2 L-type calcium channels. Proc Natl Acad Sci U S A 105: 2157–2162. 18250309 10.1073/pnas.0710501105 PMC2538892

[40] Geschwind DH (2008) Autism: many genes, common pathways? Cell 135: 391–395. 18984147 10.1016/j.cell.2008.10.016 PMC2756410

[41] Folstein SE, Rosen-Sheidley B (2001) Genetics of autism: complex aetiology for a heterogeneous disorder. Nat Rev Genet 2: 943–955. 11733747 10.1038/35103559

[42] Leblond CS, Heinrich J, Delorme R, Proepper C, Betancur C, et al (2012) Genetic and functional analyses of SHANK2 mutations suggest a multiple hit model of autism spectrum disorders. PLoS Genet 8: e1002521. 22346768 10.1371/journal.pgen.1002521 PMC3276563

[43] Abrahams BS, Geschwind DH (2008) Advances in autism genetics: on the threshold of a new neurobiology. Nat Rev Genet 9: 341–355. 18414403 10.1038/nrg2346 PMC2756414

[44] Ben-David E, Shifman S (2012) Networks of neuronal genes affected by common and rare variants in autism spectrum disorders. PLoS Genet 8: e1002556. 22412387 10.1371/journal.pgen.1002556 PMC3297570

[45] Skafidas E, Testa R, Zantomio D, Chana G, Everall IP, et al. (Sept 11, 2012) Predicting the diagnosis of autism spectrum disorder using gene pathway analysis. Mol Psychiatry: 10.1038/mp.2012.1126. 10.1038/mp.2012.126 PMC3966080 22965006

[46] Bader PL, Faizi M, Kim LH, Owen SF, Tadross MR, et al (2011) Mouse model of Timothy syndrome recapitulates triad of autistic traits. Proc Natl Acad Sci U S A 108: 15432–15437. 21878566 10.1073/pnas.1112667108 PMC3174658

[47] Liao P, Soong TW (2010) CaV1.2 channelopathies: from arrhythmias to autism, bipolar disorder, and immunodeficiency. Pflugers Arch 460: 353–359. 19916019 10.1007/s00424-009-0753-0

