# Supplementary material for: Rare Mutations of CACNB2 Found in Autism Spectrum Disease-Affected Families Alter Calcium Channel Function
Source: PLoS One. 2014 Apr 21;9(4):e95579. doi: 10.1371/journal.pone.0095579 (PMC3994086; doi:10.1371/journal.pone.0095579)
Supplement: Table S1 — All found variants in the exons and flanking intronic regions of CACNB2 in autistic patients. (DOC) [file pone.0095579.s001.doc]

**Table S1.** All found variants in the exons and flanking intronic regions of CACNB2 in autistic patients.

| **SNP ID** | **Position (build 37)** | **Position cDNA** | **Closest exon** | **Function type** | **MAF****ASD** | **MAF** **Con** | **MAF****dbSNP** | **PolyPhen2 score** |
| --- | --- | --- | --- | --- | --- | --- | --- | --- |
| rs138094231 | 18429624 | NM_201597.2: c. 1-42C>A | Exon 1a | 5’-UTR | 0.042 | - | 0.036 |  |
| New 1 | 18429646 | NM_201571.2: c.1-128C>G | Exon 1a | 5’-UTR | 0.003 | - | x |  |
| rs12572321 | 18430220 | NM_201571.2: c.36+76G>A | Exon 1b | Intronic | 0.357 | - | 0.107 |  |
| New 2 | 18430221 | NM_201571.2: c.36+77C>A | Exon 1b | Intronic | 0.003 | - | x |  |
| rs951045 | 18550270 | NM_000724.3: c.48+24T>C | Exon1c | Intronic | 0.010 | - | x |  |
| New 3 | 18629821 | NM_201590.2: c1-35G>A | Exon 1d | 5’-UTR | 0.008 | - | x |  |
| rs3750592 | 18691018 | NM_201597.2: c.168+46G>A | Exon 3 | Intronic | 0.531 | - | 0.389 |  |
| New 4 | 18691019 | NM_201597.2: c.168+47C>A | Exon 3 | Intronic | 0.003 | - | x |  |
| rs11014511 | 18787195 | NM_201597.2: c.334-89T>C | Exon 4 | Intronic | 0.117 | - | 0.077 |  |
| rs12257065 | 18787448 | NM_201597.2: c.291+42C>G | Exon 4 | Intronic | 0.003 | - | 0.005 |  |
| New 5 | 18789665 | NM_201597.2: c.292-76G>A | Exon 5 | Intronic | 0.013 | 0.012 | 0.014 |  |
| rs4485000 | 18789724 | NM_201597.2: c.292-17T>G | Exon 5 | Intronic | 0.282 | 0.384 | 0.310 |  |
| **New 6** | **18789784** | **NM_201597.2:c.334G>A** | **Exon 5** | **missense p.G167S** | **0.003** | **0.000** | **x** | **PrD 0.985** |
| **rs150528041** | **18789875** | **NM_201597.2: c.425C>T** | **Exon 5** | **missense p.S197F** | **0.003** | **0.000** | **0.001** | **PD 0.942** |
| New 7 | 18795357 | NM_201597.2: c.593-43G>A | Exon 6 | Intronic | 0.003 | - | x |  |
| rs12357063 | 18803338 | NM_000724.3: c.639+40A>G | Exon 7a | Intronic | 0.084 | - | 0.057 |  |
| **New 8** | **18803956** | **NM_201597.2: c.553T>C** | **Exon 7c** | **missense p.F240L** | **0.003** | **0.000** | **x** | **B 0.0** |
| New 9 | 18806874 | NM_201597.2: c.627C>G | Exon 8 | Synonymous p.G264G | 0.035 | - | x |  |
| rs138423466 | 18806883 | NM_201597.2: c.636G>A | Exon 8 | Synonymous p.L267L | 0.013 | - | 0.004 | x |
| rs76956014 | 18816564 | NM_201597.2: c.756G>A | Exon 10 | Synonymous p.S308S | 0.007 | - | 0.009 | x |
| rs4314963 | 18816633 | NM_201597.2: c.817+7C>T | Exon 10 | Intronic | 0.154 | - | 0.220 |  |
| rs10764582 | 18823279 | NM_201597.2: c.1134+123T>A | Exon 11 | Intronic | 0.376 | - | 0.428 |  |
| rs12767496 | 18825177 | NM_201597.2: c.1230+52T>C | Exon 12 | Intronic | 0.239 | - | x |  |
| rs2272274 | 18826974 | NM_201597.2: c.1066-135T>C | Exon 13 | Intronic | 0.392 | - | 0.421 |  |
| New 10 | 18827088 | NM_201597.2: c.1066-21C>A | Exon 13 | Intronic | 0.003 | - | x |  |
| rs2228645 | 18828371 | NM_201597.2: c.1629C>T | Exon 14 | Synonymous p.Y543Y | 0.160 | 0.161 | 0.181 | x |
| New 11 | 18828446 | NM_000724.3: c.1704C>A | Exon 14 | missense p.D568E | 0.003 | 0.000 | x | B 0.175 |
| rs61733968 | 18828486 | NM_201597.2: c.1744C>G | Exon 14 | missense p.R582G | 0.020 | 0.022 | 0.013 | PD 0.999 |
| rs58225473 | 18828635 | NM_201597.2: c.1893T>G | Exon 14 | missense p.D631E | 0.154 | 0.181 | 0.148 | PD 1.0 |
| rs4747352 | 18828663 | NM_201597.2:c.1921T>G | Exon 14 | 3’-UTR | 0.235 |  | 0.236 |  |
| rs58830289 | 18828670 | NM_201597.2: c.1928T>C | Exon 14 | 3’-UTR | 0.157 |  | 0.144 |  |

**SNP**, Single Nucleotide Polymorphism; **MAF**, minor allele frequency (MAF source 1000 Genomes, NHLBI ESP or CSAgilent); **ASD**, autism spectrum disorder; **Con**, controls; **dbSNP**, NCBI database of short genetic variations; **PolyPhen2**, Polymorphism Phenotyping v2**x**, unknown; **B**, benign; **PD**, possibly damaging; **PrD** Probably damaging.
