# Supplementary material for: Rare Mutations of CACNB2 Found in Autism Spectrum Disease-Affected Families Alter Calcium Channel Function
Source: PLoS One. 2014 Apr 21;9(4):e95579. doi: 10.1371/journal.pone.0095579 (PMC3994086; doi:10.1371/journal.pone.0095579)
Supplement: Table S2 — Current and voltage parameters (mean ± SEM) of the constructs tested. P-values stem from individual Student t-tests of the indicated channels. (DOC) [file pone.0095579.s002.doc]

**Table S2:** Current and voltage parameters (mean ± SEM) of the constructs tested. P-values stem from individual Student t-tests of the indicated channels.

|  | **-β2d** | **β2d_WT** | **β2d_G167S** | **β2d_S197F** | **β2dE7c_WT** | **β2dE7c_F240L** | **p-values, unpaired t-test** | | |
| --- | --- | --- | --- | --- | --- | --- | --- | --- | --- |
|  | **N=3** | **N=9** | **N=11** | **N=8** | **N=5** | **N=5** | **β2d_G167S vs. β2d_WT** | **β2d_S197F vs. β2d_WT** | **β2dE7c_F240L vs. β2dE7c_WT** |
| Peak Current Density [pA/pF] | -1.2 | -56.5 | -72.3 | -68.8 | -17.8 | -21.1 | 0.269 | 0.358 | 0.097 |
|  | ±1.0 | ±8.6 | ±10.3 | ±9.7 | ±5.8 | ±3.6 |  |  |  |
| V0.5_act_ [mV] | - | -11.9 | -9.8 | -6.3 | -13.6 | -18.7 | 0.339 | 0.099 | 0.054 |
|  |  | ±2.0 | ±1.2 | ±2.5 | ±0.36 | ±2.5 |  |  |  |
| **Steady-state Inactivation** |  | **N=7** | **N=4** | **N=6** | **N=2** | **N=5** |  |  |  |
| V0.5_inact_ [mV] |  | -38.7 | -47.9 | -35.7 | -33.3 | -28.2 | 0.083 | 0.456 | 0.181 |
|  |  | ±1.8 | ±5.6 | ±3.7 | ±0.7 | ±1.9 |  |  |  |
| dV |  | 9.7 | 16.9 | 13.5 | 8.1 | 8.8 | *** 0.025** | *** 0.038** | 0.650 |
|  |  | ±1.2 | ±2.9 | ±1.0 | ±1.5 | ±0.7 |  |  |  |
| **% Inactivation**  **after 150ms** |  | **N=9** | **N=11** | **N=6-8** | **N=5** | **N=5** |  |  |  |
| At -20mV |  | 49.7 | 27.6 | 23.0 | 23.9 | 42.1 | *** 0.011** | *** 0.017** | 0.113 |
|  |  | ±7.2 | ±4.0 | ±6.8 | ±2.9 | ±9.8 |  |  |  |
| At -10mV |  | 56.3 | 45.3 | 25.1 | 32.7 | 51.0 | 0.270 | *** 0.015** | 0.077 |
|  |  | ±8.9 | ±4.9 | ±1.7 | ±4.6 | ±7.7 |  |  |  |
| At 0mV |  | 61.4 | 51.6 | 29.8 | 37.4 | 56.1 | 0.306 | **** 0.003** | 0.066 |
|  |  | ±8.0 | ±5.3 | ±3.1 | ±4.5 | ±7.5 |  |  |  |
| At 10mV |  | 67.8 | 55.9 | 32.9 | 43.0 | 75.4 | 0.279 | **** 0.003** | *** 0.045** |
|  |  | ±9.4 | ±5.9 | ±1.5 | ±6.5 | ±12.0 |  |  |  |
| **% Inactivation**  **after 1000ms** |  | **N=6** | **N=4** | **N=6** |  |  |  |  |  |
| At 0mV |  | 93.9 | 98.6 | 96.0 |  |  | 0.071 | 0.513 |  |
|  |  | ±1.3 | ±2.0 | ±2.8 |  |  |  |  |  |

**V0.5_act_,** Voltage of half-maximal activation; **V0.5_inact_,** half-maximal inactivation voltage, dV, slope factor; **-β2d,** mock transfected cells; * indicates P<0.05 and ** indicate P<0.01 versus corresponding WT.
